# Off the wall

**DOI:** 10.7554/eLife.05427

**Published:** 2014-11-26

**Authors:** Dennis Claessen, Gilles P van Wezel

**Affiliations:** 1 **Dennis Claessen** is in the Institute of Biology, Leiden University, Leiden, Netherlands; 2 **Gilles P van Wezel** is in the Institute of Biology, Leiden University, Leiden, Netherlands and Microbial Ecology, NIOO-KNAW, Wageningen, Netherlandsg.wezel@biology.leidenuniv.nl

**Keywords:** cell wall-free bacteria, cell division, L-forms, *S. aureus*, *C. glutamicum*, bacterial proliferation, *B. subtilis*, *E. coli*, other

## Abstract

Bacteria that grow and proliferate despite having been stripped of their cell wall may provide insights into how primordial cells could have propagated billions of years ago.

**Related research article** Mercier R, Kawai Y, Errington J. 2014. General principles for the formation and proliferation of a wall-free (L-form) state in bacteria. *eLife* **3**:e04629. doi: 10.7554/eLife.04629

**Image** A technique has been developed that allows several distinct species of bacteria (left) to grow without their cell walls (right)

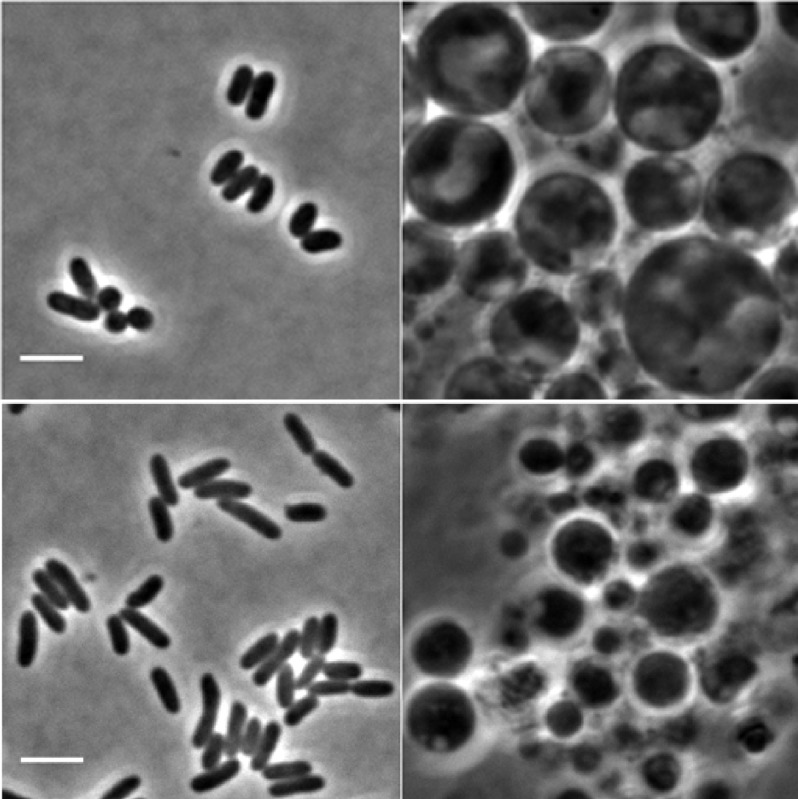

One of the most intriguing events in the history of life on earth is the evolution of self-replicating cells. Indeed, it is dazzling to even think about the complexity of the evolutionary changes required to go from a random mixture of molecules to highly organized bacterial cells that are capable of replicating themselves. A predominant theory is that RNA may have formed the basic genetic element in primordial cells and also catalysed reactions, only later to be followed by DNA and proteins (Gilbert, 1986; Orgel, 2004). Encapsulating these molecules inside a single structure would have been a key step towards creating life. However, many important questions are still a matter of debate. How did the primordial cells become encapsulated? How did these primitive life forms proliferate in the absence of proteins? And how did the molecular machinery needed for cell division emerge?

Now, in *eLife*, Romain Mercier, Yoshikazu Kawai and Jeff Errington at the University of Newcastle show that in the absence of their cell wall, different classes of bacteria proliferate using common principles (Mercier et al., 2014). Such cells can proliferate without the molecular machinery that is usually needed for cell division and cell-wall synthesis (Figure 1), thereby providing insights into what ancient microbial life could have looked like.

One of the defining structures of modern bacteria is their cell wall, which is predominantly composed of a polymer of sugars and amino acids called peptidoglycan (PG). It was demonstrated around 80 years ago that many bacteria can grow without a structural cell wall: these bacteria are called L-forms because they were first discovered in the Lister institute (Klieneberger, 1935). Classically, L-forms were isolated by cultivating cells in solutions containing high levels of chemicals that inhibit the synthesis of peptidoglycan, and lytic enzymes that break down cell walls (Allan, 1991). However, the difficulties associated with this method have meant that a consensus regarding how L-forms grow and proliferate has so far remained elusive.

Mercier, Kawai and Errington now show that common principles for L-form proliferation exist in Gram-positive and Gram-negative bacteria. Previous work focused on the Gram-positive *Bacillus subtilis* (Mercier et al., 2013); now they have now generated L-forms in the Gram-positive bacteria *Staphylococcus aureus* and *Corynebacterium glutamicum*, as well as in the Gram-negative *Escherichia coli*. L-form growth could be readily realised in these bacteria by using antibiotics that specifically block early steps in cell-wall precursor synthesis (Mercier et al., 2014).

Despite these bacterial species diverging billions of years ago, their L-forms proliferate in very similar ways. In all the bacteria examined by Mercier et al., L-form proliferation occurred independently of the cell division machinery normally used by bacteria, which involves a protein called FtsZ forming a contractile ring (Bi and Lutkenhaus, 1991). Mercier et al. demonstrated this FtsZ-independence in *E. coli* by using an unstable plasmid carrying an extra copy of the *ftsZ* gene. In the presence of this plasmid, the wild-type *ftsZ* gene could be deleted, after which growth in the walled state became dependent on the plasmid. However, when L-form growth was selected for in these cells, the plasmid was readily lost; in other words, FtsZ had become dispensable for cell proliferation. The same concept applied to the L-forms of *C. glutamicum* and *S. aureus*, which is consistent with the idea that the underlying principles of L-form proliferation have been conserved among bacterial species that diverged more than 2 billion years ago (Errington, 2013; Mercier et al., 2014).

L-forms can also be prepared from the *Streptomyces* genus of bacteria (Gumpert, 1982; Innes and Allan, 2001). Streptomycetes themselves are multicellular (Claessen et al., 2014), and L-forms derived from them are very similar to those derived from unicellular bacteria. This loss of multicellular growth in the L-form suggests that having a cell wall was a prerequisite for the development of multicellularity.

Although blocking peptidoglycan precursor synthesis helps L-form bacteria to form, previous work on *B. subtilis* showed that increased cell membrane synthesis is in fact crucial for L-form proliferation (Mercier et al., 2013). Moreover, like in *B. subtilis*, reducing the amount of membrane synthesised in *E. coli* and *C. glutamicum* prevented the proliferation of L-forms. The strict correlation between the reduced production of the peptidoglycan precursors and the increased rate of membrane synthesis in L-forms is puzzling.

Mercier et al. recently proposed that the increase in membrane synthesis causes the cells to have an abnormal surface-area-to-volume ratio (Mercier et al., 2013). This may drive shape deformation and scission events that generate smaller progeny cells with more thermodynamically favourable surface-area-to-volume ratios. Taken together, these results indicate that to support L-form growth and proliferation in a wide range of bacteria, it is essential that the cells increase the amount of cell membrane they produce.

Overall, Mercier, Kawai and Errington have found that the L-forms of unrelated bacteria proliferate in a similar manner, which is independent of the complex machinery used by modern bacteria when they proliferate. This reinforces the idea that L-forms might resemble the early life forms that existed before the invention of the cell wall, which has contributed to them being the most diverse and successful organisms on the planet. Thus L-form biology allows us to further improve our understanding of the origin of bacterial life. At the same time, work on L-forms provides food for thought in terms of what is really essential for life: indeed, scientists searching for the ‘minimal genome’ might need to ask themselves if it really does need to include genes for cell-wall synthesis and cell division.

**Figure 1.**
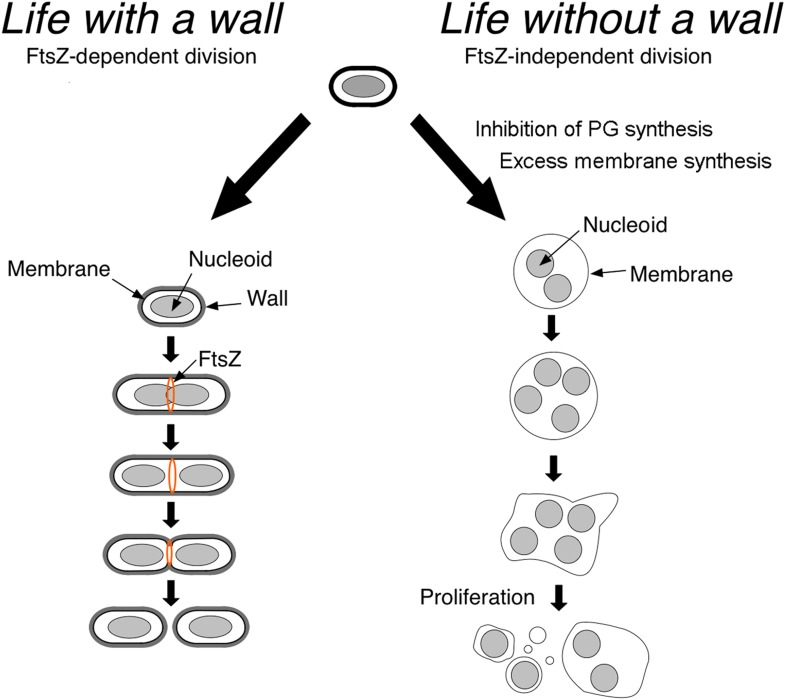
Bacteria divide in different ways depending on whether or not they have a cell wall. In bacteria with a cell wall (left), a protein called FtsZ must accumulate and form a ring that contracts to divide the cell. However, Mercier et al. found that this process is not required for bacteria without cell walls (L-forms) to divide: instead, the division of such cells is explained by physical principles that follow from the inhibition of peptidoglycan (PG) synthesis and increased cell membrane production.

## References

[bib1] Allan EJ 1991 Induction and cultivation of a stable L-form of *Bacillus subtilis*. Journal of Applied Bacteriology 70:339–343. doi: 10.1111/j.1365-2672.1991.tb02946.x. 1905284

[bib2] Bi LF Lutkenhaus J 1991 FtsZ ring structure associated with division in *Escherichia coli*. Nature 354:161–164. doi: 10.1038/354161a0. 1944597

[bib3] Claessen D Rozen DE Kuipers OP Søgaard-Andersen L van Wezel GP 2014 Bacterial solutions to multicellularity: a tale of biofilms, filaments and fruiting bodies. Nature Reviews Microbiology 12:115–124. doi: 10.1038/nrmicro3178. 24384602

[bib4] Errington J 2013 L-form bacteria, cell walls and the origins of life. Open Biology 3:120143. doi: 10.1098/rsob.120143. 23303308 PMC3603455

[bib5] Gilbert W 1986 Origin of life: the RNA world. Nature 319:618. doi: 10.1038/319618a0.

[bib6] Gumpert J 1982 Growth characteristics and ultrastructure of protoplast type L-forms from streptomycetes. Zeitschrift für Allgemeine Mikrobiologie 22:617–627. doi: 10.1002/jobm.3630220903. 6299016

[bib7] Innes CM Allan EJ 2001 Induction, growth and antibiotic production of *Streptomyces viridifaciens* L-form bacteria. Journal of Applied Microbiology 90:301–308. doi: 10.1046/j.1365-2672.2001.01243.x. 11298223

[bib8] Klieneberger E 1935 The natural occurrence of pleuropneumonia-like organisms in apparent symbiosis with *Streptobacillus moniliformis* and other bacteria. Journal of Pathology and Bacteriology 40:93–105. doi: 10.1002/path.1700400108.

[bib9] Mercier R Kawai Y Errington J 2013 Excess membrane synthesis drives a primitive mode of cell proliferation. Cell 152:997–1007. doi: 10.1016/j.cell.2013.01.043. 23452849

[bib10] Mercier R Kawai Y Errington J 2014 General principles for the formation and proliferation of a wall-free (L-form) state in bacteria. eLife 3:e04629. doi: 10.7554/eLife.04629. PMC4244569 25358088

[bib11] Orgel LE 2004 Prebiotic chemistry and the origin of the RNA world. Critical Reviews in Biochemistry and Molecular Biology 39:99–123. doi: 10.1080/10409230490460765. 15217990

